# Long-Term Clinical Outcomes of Invasive Giant Prolactinomas after a Mean Ten-Year Followup

**DOI:** 10.1155/2016/8580750

**Published:** 2016-11-23

**Authors:** Ze Rui Wu, Yong Zhang, Lin Cai, Shao Jian Lin, Zhi Peng Su, Yong Xu Wei, Han Bing Shang, Wen Lei Yang, Wei Guo Zhao, Zhe Bao Wu

**Affiliations:** ^1^Department of Neurosurgery, First Affiliated Hospital of Wenzhou Medical University, Wenzhou 325000, China; ^2^Department of Neurosurgery, Ruijin Hospital, Shanghai Jiao Tong University School of Medicine, Shanghai 200025, China

## Abstract

*Objective*. The aim of this study is to observe clinical outcomes after more than ten years of followup in a group of patients with invasive giant prolactinomas (IGPs) treated with dopamine agonists (DAs).* Methods*. Twenty-five patients met the criteria of IGPs, among which 16 patients primarily received bromocriptine (BRC) and the other nine had undergone unsuccessful microsurgery prior to BRC treatment.* Results*. After a mean follow-up period of 135.5 ± 4.7 months, the clinical symptoms in all patients improved by different degrees. Tumor volume was decreased by a mean of 98.6%, and the tumors of 19 patients had almost completely disappeared. The mean duration of treatment at maximal doses of BRC was 48.5 months. At the last follow-up visit, nineteen patients had normal PRL levels, and 14 of these patients had received the low-dose BRC treatment (at an average of 2.9 ± 0.3 mg/d). Younger patients < 25 years had a significantly higher rate of persistent hyperprolactinemia after long-term BRC treatment (*p* = 0.043).* Conclusion*. DAs are a first-line therapy for IGPs because they can effectively achieve long-term control in both shrinking tumor volume and normalizing the PRL level, and majority of patients need low-dose DA maintenance. Younger patients are prone to persistent hyperprolactinemia despite long-term DA treatment.

## 1. Introduction

Prolactinoma is the most common type of pituitary adenoma. Dopamine agonists (DAs), such as bromocriptine (BRC) and cabergoline (CAB), are the first-line treatment and effectively suppress prolactin (PRL) hypersecretion, reduce tumor size, and restore gonadal function [1–6]. DA's effect is not only positively correlated with dopamine 2 receptor (D2R) but also related to the expression ratio of the two isoforms of D2R on the surface of prolactinoma cells [7, 8]. Invasive giant prolactinomas (IGPs) are a rare subtype of prolactinomas whose criteria include a maximum tumor diameter greater than 40 mm, a serum prolactin concentration higher than 200 ng/mL, and tumor invasion into the cavernous sinus to an extent corresponding to Grade III or IV according to the classification scheme of Knosp and colleagues [9]. IGPs have an incidence rate of 0.5%–4.4% in pituitary adenomas [10, 11]. Because of its aggressive clinical behavior, the biochemical and tumor volume control in this subtype is a significant challenge [12–14].

There are a few data regarding the treatment outcomes of IGPs; however, large series are limited, and evidence-based recommendations of DAs are therefore lacking, especially for long-term treatment strategy. We previously reported 20 patients with IGPs who were treated with BRC and had a mean follow-up period of 37.3 months; we found that BRC was effective as a first-line therapy for this subtype of prolactinomas, with a mean tumor volume decrease of 93.3%. However, clinical outcomes after more than ten years of followup are largely unknown for DA-treated IGPs. In our study, we investigate the clinical outcomes after more than ten years of followup in a group of patients with IGPs treated with DAs.

## 2. Methods

### 2.1. Patient Population and Inclusion Criteria

Consistent with our previous report [2], the criteria to qualify for study participation included (1) a maximum tumor diameter greater than 4 cm invading the cavernous sinus to an extent corresponding to Grade III or IV, according to the classification scheme of Knosp and colleagues [9]; (2) a serum PRL level greater than 200 ng/mL [2]; and (3) clinical signs of hyperprolactinemia and mass effect. If patients underwent microsurgery before DAs treatment, these patients met the diagnostic criteria for IGPs according to postoperative MR imaging and endocrinological examination data and were therefore included in our study. To determine the outcomes of DAs treatment, tumor volume was calculated based on postoperative MR images in the same manner as the volume before DAs treatment.

From August 1998 to September 2007, twenty-five consecutive patients with IGPs invading the cavernous sinus were treated at our hospitals. In our previous report of 20 patients with IGPs, eight patients were lost to followup and were thus excluded from the study [2]. Seven patients were also reported in our other previous studies [13, 15]. This study was approved by the Ethics Committee of Shanghai Jiao Tong University School of Medicine and Wenzhou Medical University, and written informed consent was obtained from all patients. The cohort consisted of 18 male and 7 female patients, whose ages ranged from 15 to 52 years (mean age 35.7 ± 2.2 years) and whose pretreatment course of the disease persisted from 69 months to 192 months (mean course 102.5 months). The detailed information regarding patients' clinical characteristics is listed in Table 1.

### 2.2. Clinical Manifestation

A visual deficit was a common symptom with an incidence of 52% (13 of 25 patients), as well as headache with an incidence of 40% (10 of 25 patients). Amenorrhea was the initial manifestation in 5 of 7 women (71%), and decreased libido was the presenting symptom in 13 men (72% of men in our cohort). The most frequent symptoms and signs are detailed in Table 2.

### 2.3. Neuroradiological and Endocrinological Evaluation

Tumor mass was evaluated with a series of MR images as previously reported [2]. The maximal tumor diameter was calculated in millimeters, and tumor volume was calculated using the following formula: *V* = *πabc*/6 where *a*, *b*, and *c* are the three diameters of the tumor. According to these baseline MR images, maximal tumor diameters ranged from 40 to 73 mm. The tumor sizes and invaded parasellar areas are listed in Table 1. Invasion of the bilateral cavernous sinuses occurred in 13 cases, and invasion of a unilateral cavernous sinus occurred in the other 12 cases.

All PRL levels were greater than 200 ng/mL before BRC treatment and exceeded 4000 ng/mL in nine cases. Due to difficulties in the laboratory, accurate PRL levels were not available in some cases. Recurrence of hyperprolactinaemia following discontinuation of BRC therapy was defined as a serum PRL level greater than the sex-specific upper limit of the normal range for the assay used [serum PRL > 16 ng/mL (men) and >25 ng/mL (women)].

### 2.4. Treatment Protocol

From this cohort, 16 patients were primarily treated with BRC, and the other nine had previously undergone microsurgery with partial tumor resection prior to BRC treatment. Six of the patients underwent adjuvant radiotherapy, and two underwent gamma knife radiotherapy.

The BRC treatment protocol was followed according to our previous methods [2]: BRC was orally administered before sleep at night at an initial dose of 2.5 mg/day and gradually increased to 7.5 mg/day within 2 to 3 weeks, which was defined as the effective treatment dose in our study. If PRL level was not normalized, the dose would be gradually increased to 15 mg/day and further increased dose was not recommended. In cases in which patients experienced adverse effects after taking BRC, only half of a tablet (1.25 mg) was subsequently administered, and the dose was slowly increased. A change to CAB treatment was made in one patient.

### 2.5. Statistical Analysis

All statistical analyses were carried out using the SPSS (Statistical Package for the Social Sciences) 17.0 statistical software package. Continuous variables were expressed as the mean ± SEM. Mean values were compared using unpaired *t*-tests, and categorical variables were compared using Fisher's exact test. The significance of various variables for persistent hyperprolactinemia was analysed using the Cox proportional hazards model in the univariate and multivariate analysis to identify which factors were independent indicators for persistent hyperprolactinemia. A two-tailed *p* value test was used with a *p* value of <0.05, considered statistically significant.

## 3. Results

### 3.1. Treatment Courses

All patients were closely followed up between 90 and 192 months after treatment (mean followup of 135.5 ± 4.7 months). Initially, two patients experienced different degrees of BRC-related side effects. A lower BRC dose was administered and gradually increased. Both patients were able to tolerate the entire dose following this strategy. Over the course of BRC administration, cerebrospinal fluid (CSF) leakage occurred in one patient (case no. 20), who subsequently underwent transsphenoidal surgery to repair the CSF leak. No cases of apoplexy occurred during BRC treatment. Panhypopituitarism had occurred in 5 patients at the last follow-up visit. No cases of diabetes insipidus were observed.

### 3.2. Effects of Treatment on Presenting Symptoms

With long-term DAs therapy, headache improved in 9 patients, depending on the extent of tumor shrinkage. Among the 13 patients with decreased libido, ten showed a clinical improvement in both libido and potency associated with the normalization of PRL, gonadotrophin, and testosterone levels. Among the 13 patients who presented with visual deficits, nine showed an improvement and four did not show signs of deterioration during the long-term followup. Galactorrhoea disappeared in the two patients who had presented with it. Amenorrhea returned to normal in two of the five female patients.

### 3.3. Effects of Treatment on Tumor Volume

After more than ten years of long-term treatment with DAs, the tumor volume decreased dramatically in all patients, with a mean reduction of 98.6 ± 0.6% (range from 88.9% to 100%, Table 1). The tumor had almost completely disappeared in 19 patients; for the other six patients, residual tumor remained in the cavernous sinus area in five cases. The mean duration of treatment at maximal doses of DA (8.9 ± 0.8 mg per day) was 48.5 ± 8.3 months (range from 3 months to 135 months).

### 3.4. Effects of Treatment on PRL Level

Resistance to BRC was defined by an absence in the normalization of PRL levels when administered in a daily dose of 15 mg for at least 3 months [8, 16, 17]. In our series, five patients were resistant to BRC treatment (cases number 3, number 11, number 12, number 20, and number 22). Patient number 12 accepted microsurgery due to BRC resistance and finally had normal PRL level. Patient number 22 changed to accept cabergoline treatment due to BRC resistance and had normal PRL level. In addition, one patient (case number 10) received high dose BRC treatment (12.5 mg/d) for 96 months, but her hyperprolactinemia was not normalized; she was referred to as the resistant case. The incidence of resistance was 24% (6/25) in our study.

At the end of followup, 20 patients were still on BRC treatment, three withdrew from BRC, and two self-discontinued BRC. Nineteen patients had normal PRL levels (the rate of PRL normalization was 76%), as shown in Table 1; one of these patients changed to CAB treatment (2 mg/wk, case number 22), one accepted effective doses of BRC treatment (7.5 mg/d, case number 6), three withdrew from BRC (cases number 13, number 19, and number 21), and the other 14 patients accepted the low-dose BRC treatment (≤5 mg/d, at an average of 2.9 ± 0.3 mg/d). BRC dose ≤ 2.5 mg/d was maintained in 10 patients. Three patients with residual tumors who received low-dose BRC treatment (cases number 2, number 14, and number 18) had normal PRL levels without clinical signs of hyperprolactinemia or mass effect.

In six patients, PRL levels exceeded 25 ng/mL, 3 of whom had more than 200 ng/mL at the last follow-up visit. Among them, four patients (cases number 3, number 10, number 11, and number 20) were resistant to BRC treatment. Two patients (case number 10 and number 11) received gamma knife radiotherapy due to having a persistent residual tumor in the cavernous sinus area. Although the tumor volume almost disappeared, the PRL levels continued to exceed 200 ng/mL during the long-term followup period. These two patients self-discontinued BRC treatment; however, no obvious evidence of tumor recurrence was observed. Patient number 24 received BRC treatment with a dose of 7.5 mg/day for 135 months; however, PRL level did not reach the normal range. Patient number 25 was initially very sensitive to BRC treatment, and the PRL level decreased to the normal range; after BRC withdrawal, the patient experienced hyperprolactinemia and tumor recurrence, and BRC therapy was readministered.

### 3.5. Predictor of Persistent Hyperprolactinemia

According to whether patients had persistent hyperprolactinemia during long-term DA treatment, patients were divided into two groups: the normal PRL group and the persistent hyperprolactinemia group. Patient number 25 was initially sensitive to BRC treatment with the normalization of PRL level, so this patient was not included in the persistent hyperprolactinemia group. Thus, the persistent hyperprolactinemia group contained five patients (cases numbers 3, 10, 11, 20, and 24).

The relationship between clinicopathologic features and persistent hyperprolactinemia in 25 patients is summarized in Table 3. There were no statistically significant associations between persistent hyperprolactinemia and gender, tumor volume before BRC treatment, final tumor volume, tumor reduction percentage, unilateral or bilateral cavernous sinus invasion, duration of followup, treatment method, or duration of maximum BRC dose. A significant correlation was observed between persistent hyperprolactinemia and age at diagnosis (*p* = 0.009), or maximum BRC dose (*p* = 0.015). To determine whether age at diagnosis was important prognostic factor for persistent hyperprolactinemia among those clinicopathologic features, univariate Cox regression analysis was used in this 25-patient cohort, which indicated that age at diagnosis was important prognostic factors for persistent hyperprolactinemia (*p* = 0.043, Table 4). Furthermore, multivariate Cox regression analysis indicated that age at diagnosis was the best predictor of persistent hyperprolactinemia (*p* = 0.043, Table 4).

Together, the results showed that age at diagnosis was the only independent prognostic factor of persistent hyperprolactinemia, and it indicated that younger patients were prone to resistance to long-term BRC treatment.

## 4. Discussion

Dopamine agonists have been demonstrated as a first-line therapy for IGPs, because they can significantly shrink tumor volume and control the PRL levels [2, 10–12, 14, 18–25]. As for giant prolactinomas, the overall tumor response rate to DA treatment was 47%–87% [10, 11, 14, 19, 20, 22, 25]. In Maiter and Delgrange's recent review [14], the overall tumor response rate for giant prolactinomas to DA treatment was 74% (65/88 evaluable cases), and the hormonal response rate was 60% (58/97 evaluable cases), with a mean followup of 37 months. Chattopadhyay et al. [22] reported that 14 patients with male giant prolactinomas weretreated with BRC, and under a maximal average BRC dose of 12.3 mg/day with a treatment duration of 32.8 months, the tumor volume obviously shrank from 48.4 to 14.9 mm^3^ (69.5%). Shrivastava and colleagues reported that in 10 cases of giant prolactinomas treated with BRC, and the tumor volume decreased by a mean of 69% during a mean follow-up period of 6.7 years [10]. In contrast, the superiority of cabergoline in terms of patient tolerability and convenience, reduction in prolactin secretion, restoration of gonadal function, and decrease in tumor volume has been convincingly demonstrated [11, 19, 20]. Acharya et al. reported ten giant prolactinomas treated with CAB; the maximal tumor diameter progressively decreased by a mean of 49%, and 7 patients achieved PRL normalization [19]. In a study by Corsello et al., CAB caused a significant decrease in both PRL levels and tumor volume (98.8% and 64.8%, resp.) in ten male patients with IGPs during a mean of 38.9 months of followup [11]. Shimon et al. reported that, in twelve male patients with giant prolactinomas following CAB treatment, the tumor diameter showed a mean maximal decrease of 47% [20]. Recently, Espinosa et al. reported 47 patients with giant prolactinomas, in which 68% cases had the normalization of PRL levels and 87% had the reduction of >50% in tumor volume after CAB treatment [25].

Obviously, these studies were conducted with a limited follow-up period. To the best of our knowledge, there are two reports that consider more than ten-year long-term clinical outcomes of giant prolactinomas. Fraioli et al. utilized multidisciplinary treatment for IGPs in two children and finally reached satisfactory control of the disease with a follow-up period of 13 and 14 years [26]. Fernandes et al. reported a giant prolactinoma with 10 years of DA treatment, whose prolactin levels decreased by 96.8% with an effective reduction in tumor size [27]. In our study, tumor volume shrank by a mean of 98.6% and the incidence of PRL normalization was 76%. The clinical outcomes of our study are better than other references, which may be explained by the longer followup and by combination treatment with surgery and/or radiotherapy. It has been reported that surgery allows a reduction of the DA dose required to normalize PRL [28, 29]. In addition, radiotherapy may have contributed to the long-term control of these 8 patients in our series [30].

In our study, age at diagnosis was the only independent prognostic factor of long-term persistent hyperprolactinemia, and younger patients with IGPs were prone to be resistant to DA treatment. It has been demonstrated that DA-resistant patients tend to be younger males with larger or more invasive adenomas [31–33]. A recent study on macroprolactinomas in children and adolescents indicated that DA resistance is associated with higher PRL levels and larger tumor sizes, which are closely linked together [34]. In our study, there was no significant difference in tumor volume at diagnosis between younger age (<25) and older age (≥25) (*p* = 0.342). One of the shortfalls of this study is that accurate PRL levels are not available in some cases before BRC treatment; thus, we do not know whether pretreatment PRL level is associated with persistent hyperprolactinemia despite long-term DA therapy. On the other hand, CAB is not available in the mainland of China; thus, as for BRC-resistant patients, just one patient changed to CAB treatment, which might affect the long-term clinical outcomes.

Once the PRL level and tumor volume were adequately controlled, the dose of DA could often be decreased incrementally and then kept at a minimal maintenance dose [35]. In our study, nineteen patients had normal PRL levels and 14 of these patients accepted the low-dose BRC treatment, with an average dose of 2.9 mg/d. The majority of 14 patients (10/14, 71%) needed only a ≤2.5 mg/day low-dose BRC maintenance. Additionally, three patients with residual tumors without clinical signs of mass effect also received low-dose BRC treatment to maintain PRL normalization. Together, in our study, 56% of the patients (14/25) received low-dose BRC treatments without relapse of tumor volume or PRL level.

## 5. Conclusions

In summary, we report the clinical outcomes of more than ten years of followup in a group of 25 patients with IGPs treated with BRC. DAs as first-line therapy can effectively achieve long-term control in both shrinking tumor volume and normalizing PRL levels. Additionally, 56% of patients just need low-dose BRC treatment to maintain the long-term normalization of PRL levels and to prevent tumor recurrence. Younger patients seem to have a poor response to BRC treatment and are prone to persistent hyperprolactinemia despite long-term BRC treatment.

## Figures and Tables

**Table 1:** Summary of characteristics in 25 patients with invasive giant prolactinomas treated with dopamine agonists.

Case number	Gender	Age	PRL ng/mL		Three dimensions *a* × *b* × *c* (mm)			Followup (month)	Treatment method	Range of tumor invasion		Maximum treatment dose		Maintenance dose (mg)
			Before Tx	After Tx^*∗*^	Before Tx^†^	After Tx^*∗*†^	% change			Pre-Tx	Post-Tx^*∗*^	dose (mg)	duration (month)	
1	M	40	3154.8	Normal	40 × 34 × 30	0	100	143	S + BRC + RT	R-CS, TV	0	7.5	12	2.5
2	M	26	>4000	Normal	55 × 52 × 38	16 × 15 × 11	97.6	192	BRC	B-CS, Cl, TV	L-CS	7.5	60	2.5
3	M	29	16808	78.9	70 × 68 × 50	30 × 14 × 25/34 × 12 × 20	92.2	144	BRC	B-CS, Cl, SS, NC, TV	B-CS	15	10	5
4	M	24	>200	Normal	40 × 57 × 35	0	100	123	BRC	B-CS	0	7.5	60	2.5
5	F	47	>200	Normal	40 × 40 × 17	0	100	131	BRC	L-CS	0	7.5	24	1.25
6	M	34	>200	Normal	42 × 48 × 30	0	100	119	S + BRC	B-CS, SS, TV	0	7.5	119	7.5
7	M	34	3821	Normal	31 × 42 × 28	0	100	112	S + BRC	B-CS	0	7.5	12	3.75
8	M	23	3814	Normal	50 × 21 × 23	0	100	92	S + BRC	B-CS	0	5	12	1.25
9	F	52	12711.8	Normal	46 × 33 × 36	0	100	105	S + BRC	R-CS	0	5	105	5
10	F	26	>200	383	52 × 50 × 38	≈0	99.9	173	BRC + GK	R-CS, Cl, IPC, R-TL	0	12.5	96	*※*
11	F	22	1100	317	42 × 20 × 20	0	100	141	BRC + GK	R-CS, TV	0	15	60	*※*
12	F	24	>200	Normal	38 × 42 × 35	0	100	122	BRC + S	R-CS	0	20	24	2.5
13	M	48	1431.4	Normal	60 × 54 × 50	0	100	142	BRC	B-CS, TV, R-TL	0	7.5	24	0
14	F	22	>200	Normal	67 × 45 × 40	32 × 12 × 16	95	136	BRC + RT	L-CS, SS, Cl, TV, LV	TV, IS	7.5	12	5
15	M	49	>8000	Normal	65 × 55 × 45	0	100	165	BRC + RT	L-CS, SS, Cl, TV, L-TL	0	7.5	30	2.5^‡^
16	M	49	>4000	Normal	63 × 40 × 45	0	100	147	BRC	B-CS, SS, Cl, TV	0	7.5	35	2.5
17	M	33	>6000	Normal	58 × 49 × 51	≈0	99.9	162	S + BRC + RT	B-CS, SS, Cl, TV, NC	0	7.5	55	2.5
18	M	50	>200	Normal	50 × 30 × 30	12 × 18 × 8	96	130	S + BRC	L-CS, TV	L-CS	5	130	5
19	F	37	>8000	Normal	73 × 60 × 54	0	100	153	BRC + RT	R-CS, SS, Cl, TV, IPC, R-TL	0	7.5	7	0
20	M	15	960	141	72 × 48 × 52	40 × 25 × 20	88.9	133	S + BRC	B-CS, SS, Cl, TV, IPC, L-TL	L-CS	15	90	10
21	M	44	>200	Normal	46 × 48 × 34	0	100	119	BRC + RT	B-CS, TV, FL	0	5	12	0
22	M	46	>500	Normal	62 × 51 × 50	0	100	90	S + BRC	R-CS, R-FL, R-TL	0	17.5	60	*ξ*
23	M	47	>200	Normal	60 × 40 × 44	0	100	138	BRC	B-CS, SS, Cl	0	7.5	25	2.5
24	M	31	>6000	207	60 × 42 × 38	≈0	99.9	135	BRC	L-CS, TV, L-TL	0	7.5	135	7.5
25^€^	M	40	>4000	76	55 × 52 × 30	14 × 15 × 20	95	140	BRC	B-CS, SS, Cl	R-CS	7.5	3	7.5

Mean		35.7					98.6	135.5				8.9	48.5	

SEM		2.2					0.6	4.7				0.8	8.3	

^*∗*^Most recent finding; M: male, F: f, Tx: treatment, S + BRC + RT: surgery + bromocriptine + radiotherapy, GK: gamma knife, R: right, L: left, B: bilateral, CS: cavernous sinus, SS: sphenoidal sinus, Cl: clivus, TV: third ventricule, IPC: interpeduncular cistern, FL: frontal lobe, TL: temporal lobe, NC: nose cavity, LV: lateral ventricule, and IS: intrasellar.

^†^Values represent those for tumor areas *a*, *b*, and *c*, respectively.

^‡^This patient suffered from severe cognitive decline due to cerebral atrophy and cerebral infarction and died on March 13, 2015.

^*ξ*^This patient changed to accept cabergoline treatment (2 mg per week) due to BRC resistance and had normal PRL level.

^€^Patient withdrew BRC treatment for 35 months. Hyperprolactinemia and tumor recurrence was observed and BRC therapy was restarted.

^*※*^Those two patients had self-discontinued therapy without evidence of tumor recurrence.

**Table 2:** Pretreatment manifestations in 25 patients with IGPs.

Pretreatment presentations	Patient number	% of patients
Visual change	13	52
Headache	10	40
Dizziness	1	4
Hypopituitarism	2	8
Cognitive decline	1	4
Decreased sexual function^†^	13	72
Amenorrhea^‡^	5	71
Galactorrhea^‡^	2	29
Nasal obstruction and hemorrhage	2	8
Axillary and pubic hair loss	5	28

^†^In males, the decreased sexual function was 13/18.

^‡^In females, the incidence of amenorrhea and galactorrhea was 5/7 and 2/7, respectively.

**Table 3:** Relations between PRL levels and clinicopathologic features in IGPs.

Clinicopathologic parameters	PRL levels		*p* value
	Normal (*n* = 20)	Hyperprolactinemia (*n* = 5)	
Age	38.4 ± 2.3	24.6 ± 2.8	0.009
Gender			0.597
Male	15	3	
Female	5	2	
Tumor volume before treatment	3.18 ± 0.4 × 10^6^	4.2 ± 1.3 × 10^6^	0.33
Final tumor volume			0.562
No remnant	16	3	
Remnant	4	2	
Tumor reduction percentage %	99.2 ± 0.39	96.2 ± 2.4	0.275
Cavernous sinus invasion			0.645
Bilateral	11	2	
Unilateral	9	3	
Duration of followup	133.05 ± 5.5	145.23 ± 7.2	0.311
Treatment strategy			0.621
BRC	12	4	
S + BRC^∮^	8	1	
Maximum BRC dose	8.1 ± 0.85	13 ± 1.45	0.015
Duration of maximum BRC dose	41.0 ± 8.5	78.2 ± 20.8	0.074

^∮^S + BRC: surgery + bromocriptine.

**Table 4:** Summary of persistent hyperprolactinemia analyses by univariate and multivariate COX regression analysis.

Variable	Univariate analysis		Multivariate analysis^&^	
	HR (95% CI)	*p* value	HR (95% CI)	*p* value
Age (≥25 y versus <25 y)	0.082 (0.007–0.929)	0.043	0.082 (0.007–0.929)	0.043
Gender (male versus female)	0.723 (0.12–4.65)	0.727		
Final tumor volume (no remnant versus remnant)	1.434 (0.23–8.97)	0.7		
Cavernous sinus invasion (bilateral versus unilateral)	0.612 (0.10–3.67)	0.59		
Treatment strategy (BRC versus S + BRC^∮^)	1.6 (0.17–15.67)	0.679		

^&^Probability for stepwise: entry = 0.05 and removal = 0.1.

^∮^S + BRC: surgery + bromocriptine.
